# Dorsal root ganglion pulsed radiofrequency using bipolar technology in patients with lumbosacral radicular pain duration ≥ 2 years

**DOI:** 10.3389/fnins.2022.1021374

**Published:** 2022-11-03

**Authors:** Qipeng Luo, Zifang Zhao, Duan Yi, Shuiqing Li, Xiaoguang Liu

**Affiliations:** ^1^Pain Medicine Center, Peking University Third Hospital, Peking University Health Science Center, Beijing, China; ^2^Department of Orthopedics, Peking University Third Hospital, Peking University Health Science Center, Beijing, China

**Keywords:** chronic pain, bipolar pulsed radiofrequency, transforaminal epidural steroid injection, lumbar disc herniation, lumbosacral radicular pain

## Abstract

**Background:**

Transforaminal epidural steroid injection (TFESI) or dorsal root ganglion pulsed radiofrequency (PRF) are alternative treatments for lumbosacral radicular pain (LSRP). This study aimed to investigate the clinical efficacy of TFESI combined with dorsal root ganglion PRF using bipolar technology to treat LSRP in patients with pain duration ≥ 2 years.

**Methods:**

This prospective single-armed cohort study included 20 patients with LSRP duration ≥ 2 years, who underwent treatment of TFESI combined with bipolar PRF. The primary outcomes included numerical rating scale (NRS) and successful treatment rate (pain relief ≥50%). The secondary outcomes included Oswestry Disability Index (ODI), patient satisfaction using the modified MacNab criteria, severe complications, hospital stay and total costs. The final follow-up was 6 months postoperatively.

**Results:**

The successful treatment rate and average pain relief at 6 months postoperatively were 80% and 73.0% ± 17.5%, respectively. The successful treatment rates in patients with and without prior intervention history at 6 months postoperatively were 77.8% and 81.8%, respectively. The mean NRS score significantly decreased from 6.5 ± 0.8 to 1.1 ± 0.7 at 2 weeks postoperatively, to 1.3 ± 0.7 at 3 months postoperatively, and to 1.7 ± 1.0 at 6 months postoperatively (all *P* < 0.001), while the mean ODI score significantly decreased from 43.5 ± 2.5 to 22.5 ± 4.3 at 2 weeks postoperatively, to 20.0 ± 3.5 at 3 months postoperatively, and to 19.5 ± 3.6 at 6 months postoperatively (all *P* < 0.001). The excellent and good patient satisfaction at 6 months postoperatively was 85%. No severe complications were observed in this cohort. The average hospital stay and total costs were 3.0 ± 0.5 days and 3.36 ± 0.77 thousand dollars, respectively.

**Conclusion:**

The treatment of TFESI combined with PRF using bipolar technology might be an alternative option to treat chronic LSRP in patients with pain duration ≥ 2 years after a failure of conservative treatments, with a favorable 6-month efficacy and inexpensive total costs. However, long-term outcomes and superiority of bipolar procedure over monopolar procedure in patients with longer pain duration should be further investigated in future studies.

## Introduction

Lumbar disc herniation (LDH) is a common degenerative spinal disease and it causes low back pain and lumbosacral radicular pain (LSRP). The incidence rate of LDH is 10–20% (Ramakrishnan et al., 2017) and it brings great medical costs and economic burdens to families and society (Tosteson et al., 2008). Recently, minimally invasive interventions have become a cost-effective option and hot direction for LDH treatments.

Transforaminal epidural steroid injection (TFESI) is one of the most common interventional options for the treatment of LDH (Manchikanti et al., 2016) and several previous studies have demonstrated positive short-term effects of TFESI in reducing lumbar radicular pain (Olguner et al., 2020; Oliveira et al., 2020), and TFESI combined with dorsal root ganglion pulsed radiofrequency (PRF) can be used to treat some complex and intractable LSRP (Karaköse Çalışkan et al., 2021; Yang et al., 2021). In addition, the preoperative duration of pain can have a significant impact on the outcomes after interventional treatments (Munjupong and Kumnerddee, 2020). However, these treatments seemed to be not very effective in some patients with a longer duration time of symptoms and some complex conditions such as multilevel LDH.

Previous studies have demonstrated that single-level bipolar pulsed radiofrequency treatment was an effective procedure for treating chronic refractory lumbosacral pain (Chang et al., 2017; Lee et al., 2018), which included patients with single-level bipolar PRF after the failure of a monopolar PRF or TFESI procedure but with pain duration mostly < 2 years (mean pain duration about 1 year). However, the clinical efficacy of TFESI combined with dorsal root ganglion PRF using bipolar technology to treat LSRP in patients with pain duration ≥ 2 years remains unclear. Therefore this prospective study aimed to investigate the clinical outcomes at 6-month follow-up after multilevel treatment of TFESI combined with bipolar PRF in patients with pain duration ≥ 2 years.

## Materials and methods

### Study design

This was a prospective single-armed cohort study (sample size = 20), which had a similar study design to a previous retrospective study with a sample size of 23 published in Pain Physician (Lee et al., 2018). All participants were admitted to our institute to receive multilevel treatments of TFESI combined with bipolar PRF between December 2020 and October 2021. This study was approved by the ethics board of the Peking University Third Hospital (No.M2018092) and the study followed the Declaration of Helsinki. All patients had signed informed consent.

### Patient selection criteria

All patients were continuously enrolled according to the patient selection criteria. The inclusion criteria were as following: (i) age > 18 years old; (ii) diagnosis of mild to moderate LDH (mild LDH - maximal extrusion no more than the anterior facet line; moderate LDH - maximal extrusion no more than the intra-facet line in MRI at disc extrusion level) with unilateral chronic LSRP and numerical rating scale (NRS) scores ≥4; (iii) pain duration ≥ 2 years; (iv) a failure of conservative treatment including medication such as NSAIDs drugs, pregabalin, vitamin B12, neurotropin etc., physical therapy, best rest and so on; (v) unwilling to have open surgery or able to tolerate the interventional surgery; (vi) willing to have the interventional surgery.

The exclusion criteria were as following: (i) failed back surgery syndrome; (ii) lumbar spinal stenosis (lumbar canal mid-sagittal diameter ≤ 10 mm with typical symptoms like intermittent claudication); (iii) Medical Research Council grade ≤ 3 in lower extremity muscle strength, and incontinence of urine and feces. (iv) spinal diseases such as spondylolisthesis, spinal scoliosis, and facet joint syndrome.

### Outcome measurements

Lumbosacral radicular pain pain intensity using a NRS was the primary outcome. NRS allows the subject to choose one number between 0 and 10 that best represents their current level of pain (0 being no pain and 10 being the most intense pain ever experienced by the subject). Pain reduction percentage was defined as the following: (preoperative NRS – postoperative NRS)/preoperative NRS *100%. Pain relief by at least 50% was defined as treatment success.

The Oswestry Disability Index (ODI) was applied to evaluate the disability, and the modified MacNab criteria were used to measure postoperative patient satisfaction. The levels of patient satisfaction were determined as the following: excellent (no discomfort, no pain, no neurological signs), good (mild discomfort, no pain, no neurological signs), fair (partial relief of pain, partial relief of neurological signs), or poor (no relief of pain, no relief of neurological signs). ODI scores of ≥50% reduction were calculated.

Severe complications including nerve injury, infection, hematoma, and reoperation (including open surgeries, interventions, and endoscopic spine surgery) were recorded during the operation or after the surgery.

### Data collection

General demographic data were collected through the electronic medical record. Pain duration in patients with prior intervention treatments was collected from the time of new onset of LSRP after the last surgery to the bipolar surgery. Postoperative data were followed up through telephone questionaries or in the outpatient setting during follow-ups. Outcome variables were assessed at 2 weeks, 3 months, and 6 months postoperatively, by an independent researcher who was not in charge of the clinical treatments of the patients.

### Surgical detail

Patients were in a prone position. Under the guidance of anteroposterior and lateral C-arm fluoroscopy, the bipolar procedure of TFESI combined with PRF was performed. A pillow was placed under the abdomen during the procedure and patients were kept awake to answer questions about sensory stimulation. A total of 5 mL of 1% lidocaine was injected into the skin entry as local anesthesia.

**FIGURE 1:**
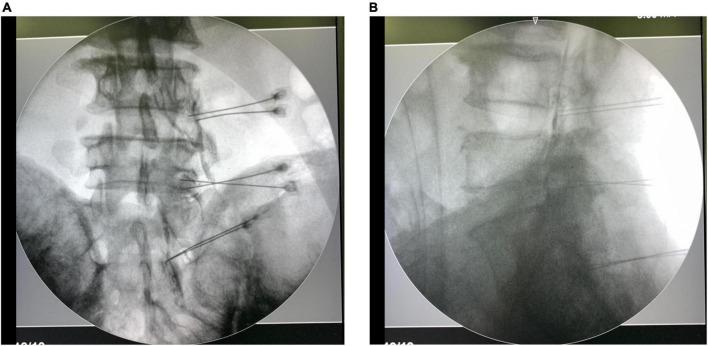
Anteroposterior of and lateral C-arm fluoroscopy images with needle placement, and spread of contrast medium at dorsal root ganglion and epidural space.

To perform the PRF procedure using bipolar technology, two 22-gauge cannulas were applied to place adjacent to the dorsal root ganglion (Figure 1). Afterward, two catheter needles with active tip electrodes were inserted through the cannulas, and the two electrodes were subsequently connected to a PRF generator at the distal end (G4, Cosman Medical, MA, USA). The distance between the 2 catheter needle tips at the same target was less than 1 cm, but they were not touching each other. In the sensory test, a voltage less than 0.5V was applied to induce tingling sensations or dysesthesia. The responsible levels were identified when the evoked symptoms covered parts of the painful area. A volume of 0.5 mL contrast medium was injected through each needle before the treatments. The PRF treatment was conducted at 42°C with a pulse width of 20 ms and a frequency of 2 Hz for 2 min, and PRF treatments in bipolar electrodes at the same dorsal root ganglion were started simultaneously.

To perform TFESI after PRF treatment, a mixture of 0.2% ropivacaine 2 mL and betamethasone about 1 mg were injected via each needle. An amount of betamethasone no greater than 7 mg was administered when multiple segments were targeted in one surgery.

The initial operation levels were roughly determined by pain areas in lower limbs and levels of LDH in MRI and CT imaging. Then the sensory test during the operation further confirmed whether the level was or not responsible for the pain. Finally, contrast medium spreading patterns were used to identify the right position of the needle tips. The next anteroposterior and lateral C-arm fluoroscopy were taken when all needles were placed or adjusted according to the last C-arm fluoroscopy.

### Postoperative management

All patients were discharged with 2-week medications, such as anti-inflammatory drugs (mainly NSAIDs), neurotrophic drugs, or neuromodulatory agents. The decisions of dose reduction, increase and withdrawal were made according to patients’ recovery during follow-ups. Patients were routinely inquired to come to outpatient clinic at 2 weeks, 3 months, 6 months postoperatively and the frequency increased if diseases worsened.

### Statistical analyses

The mean ± standard deviation or median (interquartile range) was used to present quantitative data, as appropriate, and frequency (percentage) was applied to describe categorical data. Paired student’s *t*-test was used to compare the changes of NRS and ODI scores between preoperation and postoperation or among different postoperative follow-up points, and chi-square test or Fisher’s exact test was used to analyze excellent and good satisfaction rates among different postoperative follow-up points. A p-value of no more than 0.05 was considered significant. All analyses were performed in R software (version 3.6.4). R software and Adobe Illustrator CC were used for figure preparation.

## Results

### General demographics

A total of 1,272 patients with low back pain or LSRP underwent minimally invasive intervention treatments from December 2020 to October 2021, 546 patients received endoscopic spinal surgeries and 726 patients underwent interventional treatments including dorsal root ganglion PRF, TFESI, disc decompression with percutaneous coblation nucleoplasty, medial branch radiofrequency ablation, or their combined treatments. Among 726 patients with interventional treatments, 20 patients (9 males and 11 females) with unilateral LSRP and LDH underwent bipolar procedures of TFESI combined with PRF. Fourteen patients (14/20, 70%) had the right sites of TFESI-PRF procedures. L4, L5, and S1 spinal nerve levels accounted for 75% of operation sites (15/20). Nine patients had a surgery history of nerve root blocks under the guidance of ultrasound (7/9) and TFESI combined with monopolar PRF (2/9). The average age of patients was 55.5 ± 4.6 years. The median and interquartile range of pain duration was 4(2.5, 5) years (ranging from 2 to 9 years). Patients presented moderate to severe radicular pain (NRS ≥ 4) at admission, with a mean baseline NRS score of 6.5 ± 0.8. The average operation time was 46.5 ± 16.8 min. Most patients gradually stop pain medications at 2 weeks – 3 months postoperatively. The general demographics are presented in Table 1.

**TABLE 1:** General demographics of participants.

Variables	All participants (*N* = 20)
Age (years)	55.5 ± 4.6
Gender (male)	9 (45%)
Pain duration (years)	4 (2.5,5)
BMI index (kg/m^2^)	21.3 ± 3.2
**Prior spinal surgery history**	
Prior nerve root blocks	7 (35%)
Prior TFESI combined with monopolar PRF	2 (10%)
**Procedure sides**	
Right	14 (70%)
Left	6 (30%)
**Levels of procedures**	
L2, L3 and L4	1 (5%)
L4 and L5	2 (10%)
L4, L5 and S1	15 (75%)
L5 and S1	2 (10%)
Operation time (min)	46.5 ± 16.8
Hospital stay (days)	3.0 ± 0.5
Hospital total costs (thousand US dollars)	3.36 ± 0.77

BMI, body mass index; TFESI, transforaminal epidural steroid injection; PRF, pulsed radiofrequency; Quantitative variables: mean ± standard deviation or median (interquartile range); Categorical variables: frequency (percentage); 1 US dollar ≈ 6.8 RMB.

### Clinical outcomes

**FIGURE 2:**
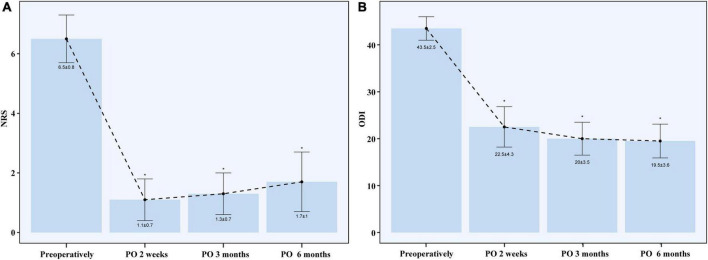
NRS **(A)** and ODI **(B)** after the operation. NRS, numerical rating scale; ODI, Oswestry Disability Index; PO, postoperative. *P value ≤ 0.001 when compared with pre-operation.

The successful treatment rate at 6 months postoperatively in this population was 80%. Two patients had NRS scores ≥ 4 at 6 months postoperatively, whose duration time of symptoms was 8 and 9 years, respectively. The average pain reduction percentage at 6 months postoperatively in these 20 patients was 73.0% ± 17.5%, and all patients achieved pain relief of more than 30% at both 2 weeks, 3 months, and 6 months after the operation. The successful treatment rates in patients with and without prior intervention history at 6 months postoperatively were 77.8 and 81.8%, respectively. Among 7 patients with prior history of nerve root blocks, two of them had pain reduction <50% at 6 months postoperatively. Two patients with prior TFESI combined with monopolar PRF both had pain relief ≥50% at 6 months postoperatively. Among 11 patients with no surgery history, two of them (18.2%) had pain reduction <50% at 6 months postoperatively. The NRS scores at the three postoperative follow-up time points were lower than those at baseline (all *P* < 0.001). There was an increasing trend of NRS scores at postoperative follow-up points from 2 weeks to 3 months postoperatively (*P* = 0.423) or from 3 months to 6 months postoperatively (*P* = 0.198) or from 2 weeks to 6 months postoperatively (*P* = 0.06), as displayed in Figure 2A. However, their average NRS scores at postoperative follow-up time points were all less than 2.

The mean ODI score decreased from 43.5 ± 2.5 preoperatively to 22.5 ± 4.3 at 2 weeks postoperatively (*P* < 0.001), 20.0 ± 3.5 at 3 months postoperatively (*P* < 0.001), and 19.5 ± 3.6 at 6 months postoperatively (*P* < 0.001), as displayed in Figure 2B. There was a decreasing trend of ODI scores at postoperative follow-up time points from 2 weeks to 3 months postoperatively (*P* = 0.172) or from 3 months to 6 months postoperatively (*P* = 0.753) or from 2 weeks to 6 months postoperatively (*P* = 0.111). Among 20 patients, 15 patients (75%) had ≥ 50% reduction in ODI at 6 months postoperatively.

The excellent and good patient satisfaction rate at 6 months postoperatively was 85%. Excellent and good patient satisfaction was achieved in 100% of patients at 2 weeks postoperatively and 90% of patients at 3 months postoperatively. There were no significant differences among 2-week, 3-month, and 6-month postoperative excellent and good satisfaction rates (*P* > 0.05), as displayed in Table 2. Two patients with NRS scores of 4 at 6 months postoperatively and with pain reduction percentages of 42.9% at 6 months postoperatively reported poor satisfaction. The other two patients with NRS scores of 3 at 6 months postoperatively and with a pain reduction percentage of 40% reported fair satisfaction at 6 months postoperatively.

**TABLE 2:** Modified MacNab criteria at postoperative different follow-up points.

Follow-up points	Modified MacNab criteria (*N* = 20)				
	Excellent	Good	Fair	Poor	Excellent/Good rates
Post-op 2 weeks	13	7	0	0	(20) 100%
Post-op 3 months	12	6	2	0	(18) 90%
Post-op 6 months	9	7	2	2	(17) 85%

Post-op, postoperative; Categorical variables: frequency (percentage).

All puncture wounds healed well without wound complications at 2 weeks postoperatively, including wound infection, pain, swelling, and redness. The average hospital stay of this cohort was 3.0 ± 0.5 days and the total hospital costs were 3.36 ± 0.77 thousand US dollars (1 US dollar ≈ 6.8 RMB). No severe complications were observed in this study.

## Discussion

In this study, we used bipolar treatments of TFESI combined with dorsal root ganglion PRF to treat LSRP in patients with pain duration ≥ 2 years. By applications of multilevel treatments, combined treatments of TFESI and PRF, and bipolar PRF procedures, which were highlights of this study, favorable 6-month outcomes were achieved in the patients whose pain duration was ≥ 2 years, with inexpensive hospital total costs and without severe complications. However, the superiority of bipolar procedure over the monopolar procedure in patients with longer pain duration should be further investigated in future studies.

Previous studies have demonstrated that bipolar PRF treatment was an effective procedure for treating chronic refractory lumbosacral or cervical radicular pain, particularly in patients whose pain is refractory to TFESI or monopolar PRF (Lee et al., 2018; Yang and Chang, 2020). In addition, bipolar PRF has better treatment outcomes when compared to monopolar PRF in Chang’s study (76 vs. 48% patients with pain relief of ≥50%) and Lee’s study (81.8 vs. 54.5% patients with pain relief of ≥50%) (Chang et al., 2017; Lee et al., 2022), which might be due to stronger electromagnetic field, and more adequately and accurately covered targets in bipolar PRF. However, the pain duration was mainly < 2 years with an average pain duration about one year, and no combined treatments (PRF alone) and single-level treatment were performed in these previous studies (Lee et al., 2018, 2022; Yang and Chang, 2020). In addition, the treatment of TFESI combined with PRF can result in greater clinical efficacy when compared to TFESI alone or PRF alone in patients with chronic LSRP (Koh et al., 2015; Ding et al., 2018; Karaköse Çalışkan et al., 2021). In addition, the degenerative spinal disease often occurs in multilevel disks, and the distribution of radicular pain is usually related to multilevel spinal nerve dermatome, especially in adults with longer pain duration (Voermans et al., 2006; Albert et al., 2019; Katsuura et al., 2020). In our study, the pain duration of all patients was ≥ 2 years (the mean duration of 4 years), which was notably longer than that in previous studies, in which the means of pain duration were about one year (Chang et al., 2017; Lee et al., 2018). The successful pain relief rate in our study was higher than that in the previous study (80 vs. 76%, 80 vs. 52.2%) (Chang et al., 2017; Lee et al., 2018), which might be related to the differences including patient selections, combined treatments (combining TFESI and PRF) (Karaköse Çalışkan et al., 2021; Yang et al., 2021), and multilevel strategy. Well, the successful treatment rate in our study was similar to that in Lee’s study (80 vs. 81.8%), which used bipolar PRF to treat refractory chronic cervical radicular pain (Lee et al., 2022).

The procedure of multilevel PRF was determined by comprehensive consideration including symptom areas, possible nerve-affected nerve root levels based on findings in MRI and CT, and intra-operative sensory test results. Although there is a cephalad and caudad flow possibility of injection solution after a single-level TFESI (Furman et al., 2012; Singh et al., 2017), PRF treatment effects can not spread to the adjacent levels. In our study, we found that a single site of dorsal root ganglion sensory stimulation could not cover all the pain complaints. Therefore multilevel PRF could be used in some complex patients such as multilevel LDH, longer pain duration, failure of single-level treatment, etc. however, prospective randomized controlled trials are needed to further confirm the superiority of multilevel PRF compared with single-level PRF.

The indications for intervention treatments and patient selections were the key points for a successful treatment. If the LSRP was caused by compression of huge herniated nucleus pulposus, severe stenosis of the spinal canal and intervertebral foramen, spondylolisthesis, scoliosis, etc., decompression surgery (open surgery or endoscopic spine surgery) was indicated in these conditions. While TFESI or PRF would be indicated for predominant neuroinflammation (Inoue and Espinoza, 2011), nerve edema, and functional alteration caused by mild or moderate LDH. In our study, we excluded the patients with failed back surgery syndrome and lumbar spinal stenosis, due to their complex mechanisms and confounders in these patients.

With the strategies including preoperative patient selection, intra-operative accurate position confirmation of the needle tip, PRF treatment of multilevel dorsal root ganglions, and administration of bipolar dorsal root ganglion PRF, the average pain reduction percentage at 6 months postoperatively was 73.0% ± 17.5% and the successful treatment rate at 6 months postoperatively was as high as 80%. However, the comparison in long-term outcomes of multilevel bipolar TFESI-PRF to TFESI alone or PRF alone or single-level TFESI-PRF is still needed to be investigated in future studies.

## Limitations

There were several limitations in this study. Firstly, although we continuously enrolled the participants for nearly 1 year and the sample size in this study (*n* = 20) was close to that in Lee’s study (*n* = 23) (Lee et al., 2018) and Chang’s study (*n* = 19) (Chang et al., 2017), the sample size was still relatively small, therefore the results from this preliminary research will be validated in our future study. Secondly, the final follow-up time was 6 months postoperatively, therefore longer follow-up is needed to evaluate long-term clinical outcomes after the bipolar treatment of TFESI combined with PRF. Thirdly, other outcomes like the 36-Item Short Form Survey (SF-36), and side effects of steroids were not investigated, which should be studied in the future. Finally, in our study, no comparison was set, therefore, the superiority of bipolar PRF over monopolar PRF in these patients with pain duration ≥ 2 years should be further investigated in future studies.

## Conclusion

The treatment of TFESI combined with PRF using bipolar technology might be an alternative option to treat LSRP in patients with pain duration ≥ 2 years after a failure of conservative treatments, even TFESI alone or PRF alone, with a favorable 6-month efficacy and inexpensive hospital total costs. However, long-term outcomes and superiority of bipolar procedure over monopolar procedure in patients with longer pain duration should be further investigated in future studies.

## Data availability statement

The original contributions presented in this study are included in the article/supplementary material, further inquiries can be directed to the corresponding author/s.

## Ethics statement

The studies involving human participants were reviewed and approved by the Ethics board of the Peking University Third Hospital (No. M2018092). The patients/participants provided their written informed consent to participate in this study.

## Author contributions

SL and XL: conceptualization, investigation, supervision, project administration, and funding acquisition. SL, XL, QL, and DY: methodology. QL, DY, and ZZ: formal analysis. QL and DY: data curation. QL: writing – original draft preparation. All authors writing – review and editing. All authors have read and agreed to the published version of the manuscript.
